# Strategies for Research, Practice, and Policy for Autism in Later Life: A Report from a Think Tank on Aging and Autism

**DOI:** 10.1007/s10803-020-04514-3

**Published:** 2020-05-02

**Authors:** Stephen M. Edelson, David B. Nicholas, Kevin P. Stoddart, Margaret B. Bauman, Laurie Mawlam, Wenn B. Lawson, Caroline Jose, Rae Morris, Scott D. Wright

**Affiliations:** 1 Autism Research Institute, 4182 Adams Avenue, San Diego, CA 92116 USA; 2 University of Calgary, Calgary, Canada; 3 The Redpath Centre and University of Toronto, Toronto, Canada; 4 Boston University School of Medicine, Boston, USA; 5 Autism Canada, Toronto, Canada; 6 Macquarie University, Sydney, NSW Australia; 7 Birmingham University, Birmingham, England; 8 Maritime SPOR SUPPORT Unit, University of Moncton, Moncton, Canada; 9 University of British Columbia, Vancouver, Canada; 10 University of Utah, Salt Lake City, USA

**Keywords:** Autism, Aging, Seniors, Think tank

## Abstract

Over the past decade, there has been a growing interest in adults on the autistic spectrum, and more recently, the challenges related to aging in this population. A two-day Think Tank meeting, focused on aging in autism, was convened amongst international leaders in the field of autism research and practice. This meeting included a series of presentations addressing the current status of aging research, followed by discussions regarding priorities going forward. Attendees shared their thoughts and concerns regarding community services, government policies, societal perspectives and physical and mental health. The goal of these discussions was to consider systematic approaches aimed at providing meaningful supports that can ensure a quality of life for seniors on the autism spectrum.

## Introduction

As people age, their physical health and cognitive abilities can be negatively impacted and, in some cases, deteriorate over time. The participants attending this meeting focused on how the aging process may affect numerous aspects of life for seniors on the autism spectrum. It is now recognized that many autistic individuals experience health and cognitive dysfunction beginning in childhood. Many of these same health and functional issues are often observed in the neurotypical population including the elderly. Some of these ongoing and emergent issues include immune and gastrointestinal disorders, reduction in bone density, sleep disorders, sensory sensitivities, weight management as well as challenges in executive and cognitive function.

The sponsors assembled participants from differing disciplines, all with relevant areas of study and expertise, and with varying clinical and research backgrounds. Given the growing identification of autistic seniors worldwide and increasing concern for their health and welfare, the goals of the meeting were to identify and address relevant clinical and research issues and to recommend best approaches to the study of age-related factors in autism.

Varied perspectives regarding preferred terminology used to describe autism were discussed at the beginning of the meeting. Two distinct perspectives included "person-first" language (e.g., “adult with autism”) versus "identity-first" language (e.g., “autistic adult”). In deference to the wishes of the autistic participants attending the meeting, the group agreed to use either identity-first language or the more neutral phrase, "adults on the spectrum."

First described in 1943, autism is a complex, life-long condition involving different etiologies, clinical presentations, and developmental trajectories. Over the years, diagnostic and clinical assessment criteria have undergone multiple iterations and adaptations (Kanner 1943; Verhoeff 2013; Wolff 2004). Autistic individuals also share commonalities including challenges in communication, social reciprocity, and behavior. Such challenges have not sufficiently been assessed throughout the life span, and little is known about how the aging process interacts with these challenges nor how autistic adults navigate and/or cope or struggle with the aging process. Such challenges likely impact overall impact experience, well-being, autonomy, life skills, and much more. Further, many autistic individuals grapple with co-occurring medical and behavioral health conditions (Ghaziuddin et al. 2002; Schiff and Asato 2015; Underwood et al. 2019); thus, inviting further focused study on autism and aging.

Historically, the focus in the autism field has been on awareness, diagnosis, and interventions, especially for children on the spectrum. Although our understanding of autistic adolescents and adults is growing, relatively little is known about autistic mid- and later-life (Wright et al. 2013). In contrast, for the general population, the fields of gerontology and geriatrics have received substantial attention, with studies and initiatives underway that may shed insight onto the aging process from the biologic, neuroscience, social science, and health service prospective (Freitas et al. 2002). Similar efforts focused on individuals on the autism spectrum will likely provide insights into providing life-long support as well as offering guidance for public policy. To that end, there is a need to review what is currently known about aging in the general population and in the autism community in order to ascertain priorities in both research and clinical and practice.

## Think-Tank Meeting/Consultation

The Think Tank meeting, held in October, 2017, was internationally sponsored by Autism Canada, the Pacific Autism Family Network, and the Autism Research Institute. Invitees included autistic adults, clinicians, service providers, researchers, and opinion leaders. The 27 participants hailed from five different countries including Canada, the United States, Australia, England and the Netherlands, the representatives from the latter two countries participating online. Although autistic individuals were present at the meeting, it was acknowledged that a greater representation of autistic individuals from the wider spectrum would have added a broader perspective on topics raised and discussed. However, every effort was made throughout the meeting to address the broad range of concerns and needs of the wider autistic community including those with severe challenges in communication and sensory sensitivities as well as those with significant medical and/or mental health issues.

The Think Tank had three objectives:

1. To bring together autistic individuals, researchers, clinicians and policy makers to identify individual and contextual factors facing the aging autistic population and their families;
2. To promote a multi-role and inter-professional approach to identifying the unmet physical, medical, social and service needs experienced by aging autistic individuals and their families;
3. To facilitate international networking and collaboration around common priorities and to advance research, knowledge, and solutions for issues related to aging in those on the autism spectrum.

## Think Tank Deliberations

Discussants extensively conveyed their perspectives about issues and perspectives about aging and autism. However, they acknowledged the need to approach this area of research, practice and policy reflection and development in sharing understanding, orientation and language. Orienting sensitivities emerged from participant reflection such that it was determined that the advancement of practice and scholarship needs to respect emergent principles of: integration and inclusiveness, proactive terminology, a socio-ecological orientation, and a focus on abilities as opposed to disabilities. Each of these orienting sensitivities are outlined below.

### Integration and Inclusiveness

It was agreed that an integrative and inclusive approach to establishing priorities involving health care, service and research agendas should include input from autistic individuals, their family members, the community, and the social-political environment. It was further determined that autistic adults should be included in formulating necessary support and interventions as well as establishing government, programmatic and policy initiatives. This perspective echoes the disability rights oriented ‘mantra’ espousing *nothing about us without us* and as such, is aligned with worldwide disability rights movements (e.g., Adoption of the Convention on the Rights of People with Disabilities [CRPD [https://www.un.org/development/desa/disabilities/convention-on-the-rights-of-persons-with-disabilities.html, 2006]). In addition, it was determined that individuals across the entire autism spectrum and their family members should have an active role in the planning and implementation of research projects.

### Socio-ecological Model

A socio-ecologic model or perspective conceptualizes autism in the context of multiple interacting factors that are viewed to be reflective of autism ‘in real life’ and more encompassing than its portrayal within a medical model. The socio-ecological model includes the person and relevant environments that interact over time, including across the life span. This model represents the need to consider aging and autism beyond the individual and includes aspects of interpersonal relationships, family, community, policy structures and the larger society and culture in which the individual resides. This conceptual model is informative for this inquiry, and has been presented in the literature as the lens used to frame the content and chapters included in the edited book and aging in autism entitled, *Autism Spectrum Disorder in Mid and Later Life* (Wright 2016).

### Abilities as Opposed to Disabilities

It was agreed that autistic individuals should be viewed in the light of their strengths and abilities, whereas their challenges should be viewed as socially constructed and not a reflection of a personal deficiency or as an anomaly or a deviation from the ‘norm’. In addition, for clarification, the term "autism community" should be used to refer to the inclusion of families and the extended community, whereas the term "autistic community" should be used to refer to the community of individuals on the spectrum.

### Emergent Themes in Supporting Knowledge, Practice and Research in Aging and Autism

Three main themes were discussed: (1) understanding how individuals on the autism spectrum progress throughout their lives, and in particular, throughout their adult lives; (2) determining how best to provide lifelong support to autistic adults and seniors, and (3) developing effective means to assess their physical and mental health using appropriate research, clinical methodologies and validated outcome measures. Each of these key themes are briefly outlined below.

### Understanding How Individuals on the Autism Spectrum Progress Throughout Their Lifetime

Several elemental considerations emerged in considering autism across the lifespan. These considerations consisted of (1) the heterogeneity of autism, (2) the scarcity and unique phenomenon of an autism diagnosis in adulthood, and (3) co-occurring medical conditions and mental health issues. Each are discussed below, as they relate to aging in autism.

#### Heterogeneity of the Autistic Population

It is acknowledged that there are varying underlying subtypes of autism. During early life, many symptoms and behaviors may be very similar and overlap, but differences may become more distinguishable and idiosyncratic over time. Because of these variabilities, autistic individuals may have different developmental and prognostic trajectories associated with variable patterns of strengths and abilities as well as challenges and vulnerabilities, any and all of which may evolve differentially over the life course. Both basic and applied research will be needed to identify, understand and differentiate these unique subtypes and processes, and to bridge the gap between biological mechanisms and clinical phenotypes and functionality.

### Diagnosis in Mid- To Late-Life

It has been established that individuals with more challenges will likely be given a diagnosis of autism early in life in contrast to those with more subtle clinical/symptomatic differences (Zwaigenbaum et al. 2019). Although our knowledge of the prevalence of autism in adulthood is limited, a United Kingdom study by Brugha et al. (2009) estimated the prevalence to be ~ 1%. However, Spencer et al. (2011) noted the potential for underestimation since “individuals with less severe disorders often go undiagnosed” (p. 891). Some autistic persons may be misdiagnosed and given a psychiatric diagnosis (Luciano et al. 2014). Volkmar and Pauls (2003) have estimated that approximately 15% of individuals on the spectrum may obtain self-sufficiency in adulthood, with an additional 15–20% able to accomplish some level of self- sufficiency with some support. Those diagnosed later in life may have milder symptoms and higher levels of functionality (Jones et al. 2014). In addition, some individuals diagnosed later in life have learned to adapt to their physical and social challenges and hence, their diagnosis may be ‘masked’. However, although undiagnosed, many of these individuals may still experience a range of autism-related and ancillary features (Griffith et al. 2012).

Unfortunately, there are as yet few validated and reliable assessments designed specifically to diagnose autism in adults (Wigham et al. 2019), and no diagnostic measures directed toward autistic seniors. Many adults and seniors on the autism spectrum have been and continue to be misdiagnosed and consequently are under-served (Au-Yeung et al. 2018). Misdiagnoses and under-diagnoses may be the result of healthcare systems in which providers are not sufficiently trained to recognize the communication, behavioral and sensory challenges as well as the medical needs and social/emotional needs faced by autistic adults. With the growing population of individuals in (and moving toward) these age-related categories, a validated assessment specifically directed to the accurate diagnosis of autistic seniors is increasingly needed in order to delineate daily and medical needs and to provide individuals access to needed and appropriate services.

#### Co-occurring Medical Conditions

In recent years, researchers have increasingly documented co-occurring medical conditions in autistic youth and adults, including allergies, gastrointestinal dysfunction, sensory-related sensitivities, urinary track disorders, pain and discomfort, dental concerns and insomnia (Davignon et al. 2018; Hohn et al. 2019). Medical conditions may also be associated with behaviors of concern, such as head banging due to migraine headaches or ear infections. Disruptive and aggressive behaviors can also be associated with pain, most especially in those who are non-verbal or hypo-verbal and who cannot express or physically indicate the presence and location of their discomfort (e.g., De Lissovoy 1962, Durand and Moskowitz 2016, Mahler 2017). Other behaviors such as eye poking has been reported to be associated with calcium deficiency (Coleman 1994). Further complicating the diagnosis of associated medical conditions is the fact that individuals may not present with symptoms typically recognized by health care providers or specialists, and may therefore be missed. More research is needed regarding the role of co-occurring medical conditions in autism, including their symptomatic presentations and behavioral manifestations, how these medical conditions and symptomatic features may change with age, and how they can be accurately identified and effectively treated. We also need to learn more about specific health conditions associated with aging, such as arthritis, cancer, hypertension, diabetes, obesity, stroke, and dementia; and how these conditions manifest in autistic individuals. In addition, we need to know how autistic individuals respond to standard therapies commonly used to treat these conditions and if different, what therapeutic approaches would prove most effective in this population.

#### Mental Health Issues

Research and proactive practice are needed to better understand and address co-occurring mental health conditions and issues (e.g., anxiety, depression, obsessive compulsive disorder (OCD), catatonia, cognitive and executive impairment), and their potential progression over the life course (Underwood et al. 2019; White and Franklin 2018). In addition, we need to understand how commonly prescribed medications, such as anti-depressants, anti-psychotics, and anti-anxiety medications, affect this population, It has been noted that some non-medical approaches may be helpful in treating some mental health issues, including cognitive-behavior therapy and mindfulness training, with some protocols being adapted to better meet the needs of autistic adults (Rodgers and Ofield 2018). Improved training of mental health professionals about how to adequately support autistic individuals should be a high priority as well as the need to significantly expand overall access to skilled mental health services within the health care system.

Several autistic participants at the ‘Think Tank’ meeting shared their personal experiences regarding the handling of traumatic events in their life. Attendees were poignantly reminded of the need to understand how autistic adults experience, process, and navigate these events in order to recommend ways to effectively manage and treat various forms of trauma (Haruvi-Lamdan et al. 2018), as well as nurture more receptive and supportive systems and communities.

Although these neuro/physiological and mental health issues comprise complex and multi-layered challenges for science and practice, greater understanding of the aging process in autism is needed in order to translate, and in some cases develop strategic interventions targeted program innovation, and efficient healthcare policies. It was noted that guidance for program and policy development should be made available in all countries in order to ensure worldwide capacity development, thereby seeking to redress resource and practice inequities that differentially limit care to autistic individuals in low to mid-income countries.

### Considerations for Developing Optimal Means to Support Autistic Adults as They Move Through Adulthood and Into Old Age

Attendees advocated for the development and implementation of evidence-based and informed supports to prepare autistic adults for a positive transition into and through old age. They identified priority aims of greater awareness and understanding, education, and employment. Each of these aims are outlined below.

#### Increase Awareness and Understanding

It was postulated that better public awareness of adults and seniors on the autism spectrum will likely lead to improvement in communication, accommodations, and opportunities for autistic adults and seniors which, in turn, may optimize their quality of life. For example, in ensuring resources to navigate one’s environment, greater consideration was advocated for an individual's style of social communication and sensory sensitivities. Besides the general public, those working directly or indirectly with autistic individuals should be made aware of the issues and challenges often faced by autistic seniors, and ways to proactively address these challenges. Among others, such groups include: (1) personnel working at residential and long-term care facilities, (2) healthcare and allied professionals, and (3) first responders (e.g., emergency services). It was particularly noted that greater awareness is needed about the variety of sensory sensitivities that exist among autistic individuals.

#### Education

Participants noted that postsecondary education in relation to employment opportunities can be viewed with both enthusiasm and concern. There remains a void in services and support for many autistic individuals following their transition from high school or college to employment (Anderson et al. 2018; Lambe et al. 2018). The capacity for learning, in general, does not end at 18 years of age (i.e., at the end of one’s formal/traditional high school education). Publicly-funded postsecondary education and professional training are needed to support autistic adults in achieving employment and career goals, and to attain and retain meaningful positions in the workforce. While receiving a postsecondary education, some autistic individuals may need support to handle various pressures of schooling and campus life, such as social relationships, studying for exams, and writing reports (Anderson et al. 2018). Moreover, ancillary supports may be needed including accessible and affordable mental health services as well as assistance in navigating time, educational requirements and community living.

#### Employment

It was noted that for adults, a stable job provides opportunities for social interaction, financial security, and independence. Employment can lead to a positive sense of well-being and a lower risk of depression (Hedley et al. 2019). However, long-term employment can be difficult even for those with training and academic credentials (Taylor et al. 2015; Baldwin et al. 2014). Unfortunately, many employers today are unwilling or unable to make the necessary accommodations to provide the required support to address autistic individuals’ unique needs. Moreover, because of the unnecessary ancillary social or sensory demands of a work environment and the lack of supports, many individuals on the spectrum remain unemployed, or are employed in jobs that are below their skill level and/or not personally rewarding (Taylor et al. 2015).

Positive attitudes and flexible business environments are needed to create more accessible and sustainable jobs. Public and private sectors need to nurture meaningful jobs for autistic individuals as well as enhance support and guidance for their career aspirations. Family and community partnerships can also play an important role in preparing individuals for employment and supporting steady and fulfilling jobs (Nicholas et al. 2018).

It is important to note that unemployment can lead to social isolation, personal struggle and financial strain, factors that can have a bearing on individual and family financial reserves and thus options for, and well-being in, senior years. Furthermore, unemployment, under-employment and/or insufficient community supports associated with employment may leave autistic adults at greater risk for depression and other mental health issues. Understanding the ongoing impact of these risk factors on the aging autistic individual warrants further study and proactive action for change.

To better understand and address key employment issues over time, there is need for longitudinal studies on employment and its impact over the lifespan, including into old age and in retirement. Such research should include methodologies and metrics to assess numerous parameters such as: (1) engagement, retention and susceptibility, (2) capacity building among employers and coworkers, (3) community involvement, and (4) the relationship of employment to what is important to the autistic adult, in the development of a desired career.

#### Community-Based Support

Meeting attendees recommended more community-based solutions and interventions to redress social isolation and promote inclusivity (Farley et al. 2018). In addition, attendees determined that there is need for more effective advocacy, with regard to supporting life course decisions and opportunities for both individuals on the spectrum and their care providers.

Rather than starting new programs focused on older autistic adults, it was thought that a place to start might be to begin to systematically examine existing programs in relevant sectors and communities, to consider how they might be adapted and expanded to meet the needs of autistic adults. It was recognized that there is a substantial knowledge base on the aging process in non-autistic individuals, which may inform or offer ‘points of exploratory departure’ when studying the aging process in autism. Research on gender difference and other social determinants may be important in attending to diverse care needs, including consideration of requisite options in care resources for elderly autistic adults.

#### Housing

There is a substantial need noted among participants to develop solutions and optimal options for community-based residential living (Mandell 2017). In general, long-term care programs for the non-autistic population is far from ideal, but similar programs for autistic individuals and others living with various neurodevelopmental conditions, likely are much more challenging. Caregivers employed by retirement homes and/or other long-term care facilities may be ill-prepared to accommodate the needs of the autistic population. Thus, specialized training programs are needed for staff in residential care facilities as well as the development of alternatives to facility-based care. Much can be learned from studying inclusive multi-generational and trans-generational housing alternatives, which are gradually becoming popular in the United States and various other world regions (Cohn and Passel 2018).

#### Opportunities for Sharing Personal Needs and Concerns

Personal concerns about aging and autism were shared by autistic adults who attended the meeting. The importance of attentively listening to autistic people ‘deeply’ and responsively was highlighted; this is always important but particularly critical as they approach and/or experience mid to later life. Attendees’ concerns in approaching old age variably included: (1) maintaining an independent lifestyle, (2) self-identifying health issues prior to requiring medical intervention, (3) learning how to (and being able to) find appropriate support when needed, and (4) remembering to take medications. Those who experience anxiety and/or depression were concerned with how these challenges might change as they age.

Employed autistic adults identified apprehension about managing post-retirement life, and the associated loss of work-related colleagues and friends. For all autistic adults, concern was raised about the possibility of receiving less support from their relatives over time. In addition, autistic attendees stressed the need for their gender identity and sexuality to be acknowledged and respected throughout their life. And finally, they noted that people, in general, should be sensitive when talking about the individual's future lifestyle, and avoid raising troubling and potentially erroneous predictions. Accordingly, hope is important and not to be diminished or deposed. Individual resilience amidst challenge which is so often demonstrated by autistic people, should not be dismissed in overly-negative accounts or predictions. Proactively addressing real issues and risk is certainly recommended, but in a strengths and hopeful ethos.

Concern was raised that, in association with the aging processes and/or shifts therein (e.g., congregate senior housing), there could be less attention paid to individual rights about one’s identity, values and preferences. Moreover, that there is substantial heterogeneity both in autism and the aging process in terms of the multi-directionality of life course trajectories. Considering and respecting human rights and preferences for aging and life values and the multidimensionality of “subjective” aging merit careful intersectional consideration relative to physical, psychological, and social domains.

### Advancing Means to Effectively Assess the Physical and Mental Health of Autistic Adults, Using Appropriate Research Methodologies and Outcome Measures

Participants affirmed that substantial advancement is needed regarding adult-based autism research. However, they stressed that careful attention would need to be directed to the relevance and benefit of that research to the autistic community. Elemental considerations were identified including research recruitment, types of studies, and outcome measures, as briefly summarized below.

#### Recruitment

A major challenge in conducting research on aging in autism is the need for recruitment of a relatively large sample size of seniors on the spectrum. This is compounded by the realization that a sizable number of autistic adults are unidentified or misidentified relative to autism. In addition, some of these individuals may not wish to have a diagnosis of autism or may not be able to access (or affordably access) a diagnosis, given system capacity limits. Others may be living in diverse or precarious circumstances (e.g., homelessness, long-term care, incarceration) that may impede or preclude confirmation of a diagnosis. Concerted attention is needed by researchers, clinicians, health care providers, social workers, policy makers, etc. to better identify autistic individuals and to ensure needed support.

Some researchers have been successful in recruiting autistic individuals and their family members by relying on innovative, meaningful, and tailor-made approaches (Nicolaidis et al. 2019). Strategies have included offering various methods of communication, (e.g., online versus paper, visual versus oral), and allowing participants to choose the format that best works for the individual. In addition, data collection meetings and settings have been offered in sensory-friendly places that include accommodations for individual preferences and needs (Nicolaidis et al. 2019). Additional possible research recruitment mechanisms could include working collaboratively with support networks that have access to adults on the spectrum (Hass et al. 2016). Nicolaidis et al. (2019) have illustrated the use of participatory research methods which offer promise in ensuring engaged research approaches that remain close to the particular needs and priorities of autistic individuals.

#### Types of Studies

There was much discussion on the advantages of conducting a large-scale longitudinal study. Such a study could systematically track a large group of autistic individuals over many decades while evaluating numerous parameters on a regular basis, such as mental health, physical health, and quality of life. It was also mentioned that repeated self-reporting by autistic individuals and their care providers may be a promising way to track development, experiences and shifts in adulthood and later life.

Such a longitudinal study could also include obtaining biological samples and eventually brain tissue. This would likely provide detailed insight into how autism progresses over the life course and improve correlations between clinical phenotypes and neurobiological mechanisms. At this time, current research involving brain tissue has been impeded by the heterogeneity among the autism spectrum disorders as well as differing clinical or functional presentations and developmental trajectories. Furthermore, there has been a lack of detail regarding an individual’s medical history, development, therapies, and educational backgrounds, thus limiting opportunities for reliable clinical and neurobiological correlations.

Unfortunately, longitudinal studies, in general, are difficult to sustain and are costly. This work, as well as the need to address research gaps relative to autism in adulthood, warrant further funding for robust and impactful studies to optimize knowledge gain. Furthermore, it will take a considerable amount of time to find answers to many pressing questions, such as optimally effective interventions. Cross-sectional research is a methodological option that allows comparison of individuals of different ages. However, since there are numerous trajectories, it would be challenging to objectively associate each cross-section to one another.

#### Outcome Measures

There was consensus among attendees that standardized and targeted metrics are needed to assess the unique symptoms of autistic adults, and to more effectively evaluate their levels of functioning. Accordingly, further metric development is needed. To that end, validated data measurement tools and methods which have been developed in the gerontology field may potentially be adaptable for autism. Given the proportionally small number of currently identified autistic adults over 50 years of age relative to younger cohorts, it was recommended that research groups worldwide combine their data to increase overall sample sizes and work collaboratively to optimize comparison controls with the aim of yielding statistically significant outcomes. The development of research collaborations will increase the likelihood of obtaining impactful population-based results in a relatively short period of time, yet attention to cultural variation across international sites may be an important methodologic and interpretive consideration.

#### Program Success Stories

Building on successes, discussants identified the importance of identifying evidence-informed and/or promising practices for older autistic adults. Identified examples of promising programs/incentives were outlined, as follows:

- The Ontario (Canada) Working Group on Mental Health with ASD (https://www.porticonetwork.ca/web/adult-asd) has developed strategic plans to increase awareness about mental health needs of autistic adults among physicians.
- The Redpath Centre in Canada (https://redpathcentre.ca/) provides various services for autistic individuals and other neurodevelopmental conditions throughout the lifespan. They also conduct research, educate stakeholders and advocate for system change.
- First Place Arizona (USA) (https://www.firstplaceaz.org/) offers supportive housing which includes a residential transition program.
- Community-based Microboard systems (https://www.velacanada.org/vela-microboards) direct support and manage funding at the individual level. This also includes advocacy for an individual’s needs in the community.
- The Star Raft model (USA) (https://thestarraft.com/) is a tool that helps build and sustain family-friendly, person-centered support networks.

These examples demonstrate increased interventional work that is addressing the needs of autistic adults. However, it is acknowledged that substantial advancement in research, policy and service delivery is needed. Services and resources specific to autistic seniors must be developed as the population ages. Family members, professional agencies, and government departments must be prepared to provide the necessary guidance and funding to properly support these individuals.

## Discussion

The Think Tank offered important priorities, considerations and cautions in the quest to advance understanding and quality of life for seniors with autism. In moving toward that end, cautions were strongly endorsed regarding core terminology, philosophical and practical understandings as well as theoretical underpinnings in practice, research and policy development. It was concluded that common practices in research, community services, policy, and societal discourses have variably and often insufficiently rendered autistic adults as central in commentary and planning about aspects pertaining to their own lives, including how autistic individuals are viewed and supported. In response, it was argued that ‘engagement as central’ needs to underpin adult and aging-based research in autism.

Key areas of further consideration in advancing research and clinical and community practice reflected the critical importance of listening to autistic individuals across the lifespan (not just during younger years), with particular attention on the heterogeneity of autism, co-occurring conditions and/or other salient issues, improved supports for aging autistic adults, and strategies to redress existing barriers to quality of life. Methodological advancement in building a strong aging autism research base has been emphatically advocated with particular attention needed in methods of recruitment, study design and data analysis. It is hoped that increased research in autism and aging will better support the autism community relative to supports and services specifically directed to autistic seniors. Applying current challenges, concerns and risks to seniors in congregate living facilities as a result of the COVID-19 pandemic warrant careful study about the unique risks and challenges faced by elderly and/or frail autistic persons in such settings and circumstances.

In addressing these substantial issues and opportunities for advancement, pioneers in this field are encouraged to collaborate in order to advance collective approaches, via visionary leadership and seeking increasing funding support from the scientific and policy communities. More expansive and sustainable collaborations are needed in the aim of finding solutions to the challenges faced by autistic seniors now and in the future. Such research and action must be linked to direct quality of life benefits via supporting optimal community-based services and resources for autistic people across the lifespan.

## Conclusion

Key areas of practice and research development relative to autism and aging were informed by this Think Tank. Listening well, better understanding and proactively responding to aging autistic individuals relative to their experiences and needs emerged as a central to action and collective advancement. Appraising the impact of autism on aging (and vice versa) merit public investment as does supporting aging autistic people in their right for, and important pursuit of, lives of quality.
